# Deep Learning Approach to Impact Classification in Sensorized Panels Using Self-Attention

**DOI:** 10.3390/s22124370

**Published:** 2022-06-09

**Authors:** Stefan Karmakov, M. H. Ferri Aliabadi

**Affiliations:** Department of Aeronautics, Imperial College London, Exhibition Road, South Kensington, London SW7 2AZ, UK; karmakovst@gmail.com

**Keywords:** structural health monitoring, impact classification, passive sensing, composite materials, deep learning, transformer, convolutional neural network

## Abstract

This paper proposes a new method of impact classification for a Structural Health Monitoring system through the use of Self-Attention, the central building block of the Transformer neural network. As a topical and highly promising neural network architecture, the Transformer has the potential to greatly improve the speed and robustness of impact detection. This paper investigates the suitability of this new network, confronting the advantages and disadvantages offered by the Transformer and a well-known and established neural network for impact detection, the Convolutional Neural Network (CNN). The comparison is undertaken on performance, scalability, and computational time. The inputs to the networks were created using a data transformation technique, which transforms the raw time series data collected from the network of piezoelectric sensors, installed on a composite panel, through the use of Fourier Transform. It is demonstrated that the Transformer method reduces the computational complexity of the impact detection significantly, while achieving excellent prediction results.

## 1. Introduction

Composite materials have gained popularity for numerous engineering application in the past two decades. Their outstanding properties, such as high specific strength, resistance to fatigue damage, and corrosion resistance, make them particularly attractive for the aerospace industry [1,2]. Yet small defects and damages can significantly deteriorate these superior mechanical properties. Defects such as delamination, matrix cracking, and fibre fracture are common in composites and can cause catastrophic failure of the components if not addressed [3]. Impact by a foreign object is a common way for the creation of such defects in composites [4]. From runway debris and tyre shrapnel, to hail or bird strikes, to dropped tools during maintenance, there are numerous occasions during the lifetime of an aircraft component when it can experience impact damage. Many of the defects due to impact are very hard to detect, as they tend to occur sub-ply, or are barely visible [5]. Non-destructive techniques (NDT), such as C-Scan Ultrasonic inspection or Radiography need to be used during ground check-up; such maintenance is time consuming and requires skilled operators and expensive apparatuses [6]. NDT is also prone to human error and inconsistencies. A more robust, faster, and cheaper method is required to allow for the detection of damage in composite components.

Structural Health Monitoring (SHM) has gained popularity, as it allows for the continuous monitoring of structures through the use of sensors. SHM gives insights about the overall state and behaviour of the structure, but more importantly about hard-to-access, or sub-surface regions [7]. Through the implementation of sensors inside composite lay-ups, knowledge can be gained on the degree of severity that an impact has had on the integrity of the composite part [4]. Such an implementation would be cheaper in terms of human labour, time, and maintenance costs, and will increase the robustness of localization and assessment of the severity of the damage.

During a single flight, all these embedded sensors would be constantly recording, gathering a large set of data. Normal data analysis and numerical method techniques would be infeasible; it would take too much computational power and fine-tuning to analyse all the signals. This calls for the use of deep learning networks [8]. In the past two decades, the field of neural networks has experienced an unprecedented popularity and growth, with numerous architectures being suggested, each specializing in different tasks. Through the use of pre-training, deep learning algorithm can learn patterns in data and use this knowledge to classify new sets of data. Deep learning networks excel at analysing large amounts of data with numerous parameters, as is the case with damage detection and severity estimation [8]. Our paper focuses on applying a Transformer, a contemporary and very promising neural network, to the problem of damage detection. To assess the suitability of the Transformer for this new context, a Convolutional Neural Network (CNN), a well known and widely used neural network for the task of damage detection, is built in parallel and used for validation.

## 2. Deep Learning for SHM

SHM is the act of detecting and interpreting changes in a structure through the use of sensors [9]. The goal of SHM is to improve the reliability of a structure, as well as extract information on how to improve the performance and reduce life-cycle costs [10]. SHM sensing techniques can be divided into two general categories, passive and active [4]. For passive sensing, the transducers attached to the structure only act as sensors, detecting and recording the response of the structure. The excitation to the system comes from an external source. The collected data allows for damage localization and characterization. For active sensing, the transducers attached to the structure act both as sensors, as well as the actuators that excite the system. This method provides more information about the system, yet it also makes the analysis more complex. The experimental data for this paper has been collected from a passive sensing technique [11]. As the purpose of the study is to improve the classification of impact energy levels from a foreign object on an aircraft composite structure, passive sensing is a more suitable option.

The impact of an object on a thin composite plate creates dynamic stress waves, known as Lamb waves, that propagate through the plate [12]. Lamb waves were discovered by Horace Lamb in 1917 [13], yet Worlton used Lamb waves for damage detection for the first time [14]. Rose summarized the potential of ultrasonic guided wave detection and Lamb wave for detecting damages [15]. These waves are a superposition of longitudinal and shear modes and can have symmetric or anti-symmetric modes [16]. Lamb waves can travel large distances in composites, despite composites having high attenuation ratios. They also propagate through the entire thickness of the plates, allowing for the detection of both surface and internal damages [17]. These properties make Lamb waves attractive for impact detection in plates. The waves can be captured using piezoelectric sensors (PZT sensors) mounted on the plates and information can be extracted from the voltage output [17]. Lamb wave propagation characteristics depend on the entry angle/direction, the excitation and the geometry of the plate. Lamb waves’ dependence on numerous variables, as well as their dispersive nature, make their propagation very complicated in anisotropic materials [17]. Clustering sensors very close reduces the complexity, but this method is not very practical. A solution widely practised in the SHM field is the use of Artificial Intelligence (AI) algorithms to extract damage information from the data [9].

AI has found application in a range of engineering disciplines, with machine learning and deep learning algorithms being used to analyse large and complex amounts of data. For impact and damage detection and localization, different algorithms and architectures have been proposed. Jia et al. used Artificial Neural Network (ANN) for fault diagnostics for rotating machinery [18]. Seno, Khodaei, and Aliabadi used ANN for impact localization, using passive sensing in composite panels [11,19]. Seno and Aliabadi also proposed a novel gradient method for impact force estimation [20], and a novel stochastic Kriging-based method for impact location and force estimation [21] for composite panels. Lin, Nie, and Ma used a deep CNN for automatic feature extraction from low-level sensor data, and achieved excellent structural damage localization, even with noisy data [22]. Saxena and Saad used a genetic algorithm for feature set selection, along with an ANN for monitoring of rotating mechanical systems [23]. Selva et al. used a Probabalistic Neural Network to locate in-plane damage in carbon fibre reinforced plates [24]. Abdeljaber proposed a 1-dimensional CNN for real-time vibration-based damage detection and localization for SHM [25]. Oliveira, Monteiro, and Filho developed a CNN for damage detection [26], achieving high accuracy. Guo et al. [27] used a CNN with multi-scale and residual learning modules for the task of damage detection for noisy and incomplete data. ANNs and CNNs have been widely used for a number of SHM applications, and due to their excellent performance, have dominated the field; little research has been undertaken into introducing novel architectures. This paper’s aim is to explore the Transformer as an alternative approach for SHM, inspired by its success in NLP applications. To the authors’ knowledge, the suitability of this architecture for SHM applications has not been investigated.

### Transformer Model

The Transformer is a type of network architecture based on the idea of a Recurrent Neural Network (RNN), but uses the Attention technique to focus on particular parts of the data [28]. Though new, the Transformer has quickly become the dominant model in the fields of Natural Language Processing and Machine Translation [29,30,31]. Recent work has also utilized the network’s potential for the field of computer vision [32]. This section gives a brief description to the Transformer architecture [28].

A standard RNN model is able to store information about past input data, called sequential memory. This memory of previous features helps it estimate what the current and future features will look like, making the network ideal for cases where the data is sequential, and previous events/data have an effect on future ones [33]. This sequential memory does not extend far back into the past, which is a shortcoming when longer times series are used. This problem was addressed by a new network architecture, the Long Short-Term Memory (LSTM) network [34]. It uses four distinct gates: forget, store, update, and output, which allow it to store important data and forget irrelevant one for very large sequences [35]. The Transformer uses Attention, a technique that enables the network to focus on features of the data that are more relevant to the given task, and disregards parts of the data that add less insights. Each sequence of the data is fed into the Transformer altogether, effectively in parallel, rather than split and fed sequentially in parts, as would be performed for RNN and LSTM. In the common example, where a Transformer would be applied for NLP, a sentence (the input) would be fed to the model altogether, not split and fed as single words. Being presented with the whole sequence allows the Transformer to apply Attention and weigh the importance of each word. The network is thus able to look back into the sequence much further than the LSTM, allowing it to extract global dependencies. Such input parallelization also decreases the network training time.

The Transformer is an encoder–decoder model. The encoder and decoder layer architectures are very similar, with the decoder layer having an additional sub-layer. It possesses a masking sub-layer, which stops it from accessing the sequence data after a particular marker. This is a crucial component for text generation and translation, yet the problem of the paper is a classification one, thus the decoder layer was not used. The encoder’s building blocks are briefly explained below, as proposed in the original Transformer paper [28]:

- **Embedding**: Before the input vector is fed into the network, it is embedded. This is useful in text applications, where the inputs are whole numbers that correspond to the key values for words in a dictionary/vocabulary. The Embedding replaces each word/key value with a unique dense vector, through the use of unsupervised learning, or look-up tables. Similar words are mapped to similar dense vectors. The sequence of *S* number of words is converted into a matrix, where each row is a dense vector (embedding) of length $d_{emb}$, representing a word. Encoding the inputs this way allows for a more consistent backpropagation during network learning.
- **Positional Encoding**: The positional encoding creates vectors of the positions of each word in the input sequence, via sine and cosine functions. After the positional vector is created, it is added to the word embedding. This ensures that the Transformer not only has information about the word, but also its position in the sequence, which allows for parallelization. The input matrix, containing embeddings with positional encoding, is fed to the first encoder.
- **Self-Attention**: The heart of the Transformer model, the Self-Attention sub-layer, weighs the relationships between each embedding in the input sequence and all other embeddings in that sequence. It selectively focuses the attention of the Transformer towards the embeddings that have the highest effect on the model performance. In order to do so, the input matrix is passed through three linear transformations, to produce three different matrices:
  (1) $$Q=XW^{Q},K=XW^{K},V=XW^{V}$$
  where *Q*, *K* and *V* are the query, key, and value matrices of column dimensions $d_{q}$, $d_{k}$, and $d_{v}$, respectively, and $X\in R^{S\times d_{emb}}$ is the input matrix. The transformation matrices, $W^{Q}$, $W^{K}\in R^{d_{emb}\times d_{k}}$ and $W^{V}\in R^{d_{emb}\times d_{v}}$, are the learnable parameters for this part of the layer and are updated during backpropagation. The scaled dot-product attention is then calculated using the equation:
  (2) $$Attention\left(Q,K,V\right)=softmax\left(\frac{QK^{T}}{\sqrt{d_{k}}}\right)V$$
  where Attention is a matrix of size $S\times d_{v}$, describing the relations between embeddings. As the queries, keys and values are calculated from the same input ( the previous layer) the Attention is called *Self-Attention*.
- **Multi-Headed Attention**: Passing the data through one Self-Attention sub-layer will extract some data dependencies. In order to boost performance and capture a larger number of relations from a sequence, *N* number of Self-Attention sub-layers (heads) can be stacked in parallel to create a Multi-Headed Attention sub-layer. *Q*, *K* and *V* matrices are created for each head independently, using learned linear projection weight matrices. The column dimensions of the query, key, and value for each head are taken to be the same, equal to $d_{N}=d_{emb}/N$. This creates different subspace representations of the query, key, and value for each Attention head, which then are fed into the scaled dot-product attention:
  (3) $$Head_{i}=Attention\left(XW_{i}^{Q},XW_{i}^{K},XW_{i}^{V}\right)$$
  where *i* denotes the head number, $W_{i}^{Q},W_{i}^{K},W_{i}^{V}\in R^{d_{emb}\times d_{N}}$ are the projection weight matrices for the *i*-th head, and $Head_{i}$ is the attention matrix of the *i*-th head [36]. The separate query, key and value representations allow each head to derive different information from the same input data. At the output of all the parallel heads, the calculated attention matrices are concatenated, to form a matrix with the dimensions of the input matrix, $S\times Nd_{N}$. This new matrix is multiplied by a matrix of weights $W^{O}\in R^{d_{emb}\times d_{emb}}$. This extracts global relations from the sequence data. It also gives one more weight matrix for the network to optimize and improve performance. Deeper analysis into Self and Multi-Headed Attention can be found in [37,38].
- **Feed-Forward Network**: The feed-forward network (FFN) sub-layer extracts features from the Multi-Headed Attention output with the intention of further summarizing the encoding process, thus its input dimension is $d_{emb}$. The output of the FFN is fed into the next encoder layer. This limits the last layer of the network to be equal to $d_{emb}$ in order for the output dimensions to be the same as the input. This ensures the next encoder layer will be able to read and manipulate the output from the previous one. In the original Transformer architecture, a two-layer network, with a ReLU activation, is used [28].
- **Add and Norm**: This operation consists of first applying a residual skip connection [39] around each of the encoder sub-layers, namely adding the input of the sub-layer to its output. This is followed by a layer normalization operation [40]. The Add and Norm is applied after the Multi-Headed Attention and the Feed-forward network sub-layers.

## 3. Research Methodology

### 3.1. Experimental Data

**Figure 1:**
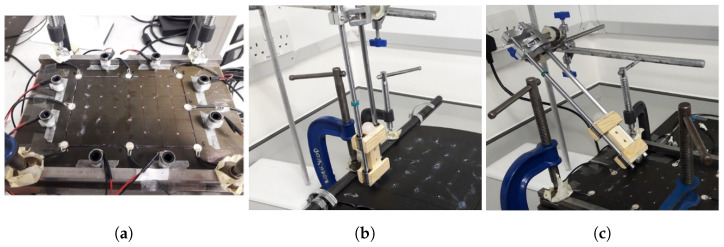
Experimental set-up of the apparatus for data collection showing (**a**): the flat composite plate, with the attached sensors, (**b**): rail guided impactor at $90^{{}^{\circ}}$, and (**c**): rail guided impactor at $45^{{}^{\circ}}$. The figure is a modified version of Figure 1 in [11].

**Figure 2:**
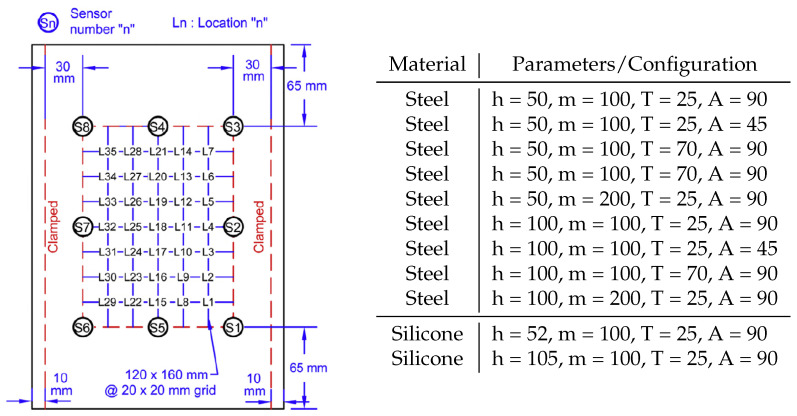
Layout of the plate tests with the sensor and impact locations. The figure is a modified version of Figure 2 in [11]. The table gives information about the configurations for the performed tests.

The data used to train and validate the network was taken from Seno, Khodaei, and Aliabadi’s work on the paper [11] on passive sensing for damage detection on composite plates. A flat composite plate, with dimensions 200 mm by 290 mm, and made of two quasi-isotropic ([0/+45/−45/90/0/+45/−45/90]s layup) carbon fibre (M21 T800s) layers was subject to impact by a 20 mm round ball placed on a rail guider. Eight PZT sensors were bonded to the impact side of the plate, on the bottom side a silicone heating mat and a temperature sensor were placed, and the plate’s long edges were clamped, as shown in Figure 1. With this set-up, the control variables/parameters were: impactor material, drop height, impactor mass, temperature, and angle. A total of 35 locations, distributed in a grid pattern, were tested, as shown in Figure 2. The test at each location was repeated 4 times for consistency, and an aggregate of 11 different configurations of impact condition were tested, amounting to a total of 1540 tests, further described in the table in Figure 2. Two materials, steel and silicone, were used for the impactor, with the impact angle being set to 90 or 45 degrees. All steel impactor tests are 1260, out of which 280 are from 45 degree impacts.

The paper’s aim is to be able to accurately classify impact energy, given knowledge of the Lamb waves induced in the impacted plate. The Lamb waves were detected and collected through the use of the mounted sensors. Knowledge of the impact energy gives an understanding of the severity of the impact; a severe impact is associated with composite damage, such as delamination and fibre cracking. The classification of impacts was performed on the basis of the potential energy of the impactor, using the formula $E_{p}=mgh$, where *m* and *h* are the impactor mass and drop height, while temperature was a control variable during the collection of the impactor data [11], it is not used for the analyses, as it does not affect the potential energy. The impactor potential energy was chosen, as it is a simple measurement, through which the impact tests can be easily and very distinctly divided into three classes:

- *Safe*: drop height-50 cm, mass-100 g/drop height-52 cm, mass-100 g;
- *Warning*: drop height-100 cm, mass-100 g/drop height-50 cm, mass-200 g/drop height-105 cm, mass-100 g;
- *Danger*: drop height-100 cm, mass-200 g.

### 3.2. Data Transformation

The control variables during the data collection were material type, impactor height and mass, temperature, and angle of contact. In order to teach a robust neural network, the network should be fed information with a distribution that encompasses all tested changes to the variables. This would create a bigger variance in the parameters, making it harder for the network to reach convergence and output accurate results. Yet overall the network would have seen data with a larger distribution, allowing it to generalize better.

**Figure 3:**
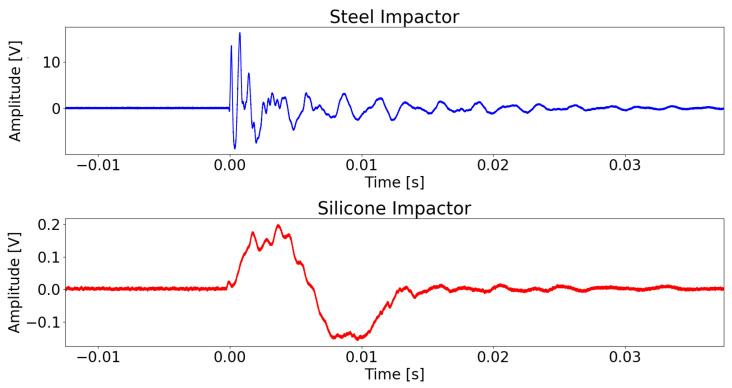
Sensor signal recording for a steel and silicone impactor. All parameters between the two impacts are kept the same, only the impactor material was changed.

The only exception to this strategy is the impactor material variable. The largest difference in signal structure is between silicone and steel impacts. The signals from the silicone impactors are not as sharp, with smaller amplitudes and lower frequencies, greatly differing from the steel impactor signals, see Figure 3. Upon impact, the softer silicone impactor would deform more, absorbing more of the impact energy. The contact area would also increase, leading to a more gradual energy transfer to the plate. This would result in the recorded Lamb waves being with a smaller amplitude and with a larger period components. It was decided that including both steel and silicone data would confuse the networks because of these discrepancies. The data transformation steps taken were performed on both the silicone and the steel data, yet only the steel data, being larger, was fed to the network. The silicone data was left for scalability testing.

**Figure 4:**
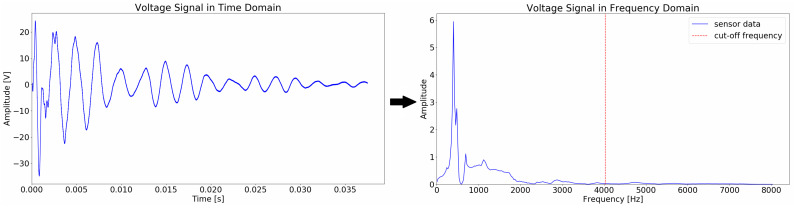
Transformation of the time series signal into a frequency domain using a Fast Fourier Transform. The red line marks the high end of the passband frequency range, with 0 Hz being the low end.

For each conducted impact test, only the transient voltage signal data was kept. A problem that was addressed was the too large signal size, namely 75,000 data points for each sensor, for each test. Feeding such large sets of data to a neural network can lead to problems. The Transformer might not properly distinguish the important features in the signals, if the signals are long, yet the data set is not sufficiently large. Additionally, it might take the network an impractically long time to process the large data. The chosen solution is to transform the time series to the frequency domain, using a Fast Fourier Transform, as shown in Figure 4. It is worth noting that a recent paper by James Lee-Thorp et al. [41] uses Fourier Transforms in place of the self-attention sub-layers to speed up the network training time. Unlike this approach, we apply the Fourier Transform to the data before it is fed into the network, which is a novel approach to the knowledge of the authors. The single-sided FFT spectrum was taken. The amplitudes of each of the frequency components was calculated as the magnitude of the complex numbers in the spectrum. A cut-off frequency is decided based on preserving the frequencies with the highest amplitudes, thus highest contributions to the signal’s form. This cut-off frequency is a hyperparameter, which is tuned based on balancing information preservation and size reduction. The frequency of 4027 Hz was taken for this set of data; it was decided that losing the frequencies higher than this will have an insignificant effect on the carried information. This significantly reduces the dimensions of the data, as the data shrinks from 75,000 data points in the time domain to a length of 152 data points in the frequency domain, equal to the retained frequency bin values.

The original voltage signals contain high frequency noise from the apparatuses, which was thought could cause distortions in the FFT spectrum. To test this assumption, the voltage signals were first filtered with a moving average filter and after with a Savitzky–Golay filter. The signals were then transformed through the FFT. The spectrum with and without the filtering was almost identical, with meaningful deviations being visible at frequencies higher than the cut off frequency chosen. It was decided that filtering the signals for the high frequency apparatus noise, before applying the FFT, would result in insignificant improvements, thus was skipped.

The Transformer model is designed to take in a matrix, where each row is a one-dimensional vector of numbers, representing a word. The embedding process is responsible for converting the word key values to the more Transformer-readable dense vectors. For this study, the data origin is not text, but voltage signals, thus it is proposed that the embedding layer, along with the positional encoding, be skipped altogether and the input be fed directly into the encoder. The encoder requires a matrix input where each row is a dense vector and the number of rows corresponds to the size of the initial input. Now that each input is not transformed through embedding and positional encoding, an artificial equivalent is proposed. A 152 × 8 matrix is constructed for each test, where each column corresponds to the amplitudes from the frequency domain for each of the 8 sensors. The sequence length *S* is 152 and the embedding dimension $d_{emb}$ is 8, where each dense vector (1 × 8), contains frequency amplitudes for each of the 8 sensors. With this configuration, the frequency domain of all the columns(sensor data) is the same. Thus, the data contains positional encoding inherently, as all the rows are arranged in an ascending frequency order.

## 4. Network Results and Performance

### 4.1. Impact Classification

This section introduces the results obtained from the trained Transformer model on impact energy classification. Following, the results are presented in conjunction with explanations about what in the results motivated the further analysis and manipulation of the data. A comparison is drawn with the results obtained from a CNN trained with the same data, with the intention of rating the performance of the Transformer. The metrics used to evaluate the models’ accuracy are F1 score and confusion matrices. A summarizing table of the two models’ performance is presented in Appendix B, Table A1.

#### 4.1.1. Transformer

**Figure 5:**
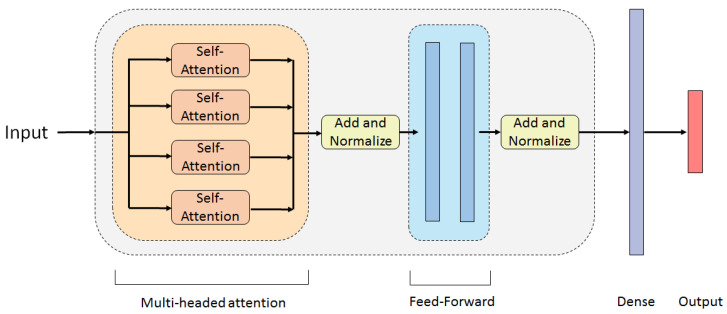
Architecture of the Transformer model.

No guidelines were found on Transformer architectures for non-text or image classification uses; a series of fine-tuning iterations were required to reach an architecture. It was decided that the standard structure of the encoder would be used, but including the aforementioned changes of removing the embedding. The architecture explained below and presented in Figure 5 is the one that yielded the best and smoothest results. A single encoder layer produced the most accurate predictions. The input matrix (152 × 8) is fed into the encoder layer, in which the first sub-layer is a multi-headed attention. Best results were achieved with four heads. For each head, the query, key, and value matrices were set to $d_{emb}/N=2$. For the linear transformations applied in the Multi-Headed Attention sub-layer, biases were added to the calculations of the query and value matrices, as well as for the calculation of the multi-headed attention, using the $W^{O}$ matrix. As an example, for a single attention head, the query is then computed by $Q_{i}=XW_{i}^{Q}+J_{S,1}b_{i}^{Q}$, where $J_{S,1}$ is a vector of ones and $b_{i}^{Q}\in R^{d_{N}}$ is the added bias for the *i*-th head, as noted in [36]. The same logic was applied to the other two linear transformations, while bias is not present in the Transformer paper [28], it improved the performance of the model used in this paper. The Feed-Forward network consists of two dense layers, each with eight nodes. A flattening layer converts the encoder output matrix into a vector, that is fed into the next dense layer. This final dense layer, outside the encoder layer, has 30 neurons that feed into the output with 3 neurons, which uses a *Softmax* activation. Each dense layer uses Leaky ReLU activations. Dropout layers [42] after each of the dense layers were also added; the dropout rate was kept low, 0.05, yet it helped the model converge quicker.

**Figure A1:**
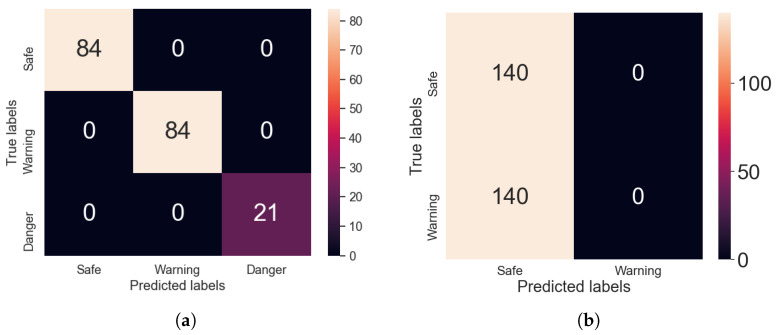
Confusion matrix on the predictions from the Transformer/CNN model, given testing data of samples from (**a**): the steel impactor test and (**b**): the silicone impactor test.

The overall tests performed with steel impactors is 1260. The tests were split into training and testing data, in a random fashion. The training set was taken to be 85% of the overall data, and the network trained on 1071 examples. The other 189 samples were reserved for testing. The proportions of Safe, Warning and Danger impacts were kept the same between the training and testing data. The Transformer model was also evaluated against the silicone impactor data. For this evaluation, the testing data comprised only of silicone impacts, a total of 280, without any steel impactor data in the testing. The F1 scores are 1 and 0.333 (see Appendix A, Figure A1). The F1 score of 1 demonstrates the perfect performance of the model, showing no misclassifications for the steel test data. The Attention technique has allowed the model to analyse the whole input and judge which parameters are most important, extracting a very accurate understanding of the data distribution. Albeit, the model was not able to distinguish between the impact energies for the silicone test data. The model was trained with both the training and testing data, with the intention of giving the model more data and increasing accuracy with the silicone classification. The silicone impact classification did not improve. This result is again attributed to the very different signal characteristics that the silicone impactors induced in the plates.

**Figure 6:**
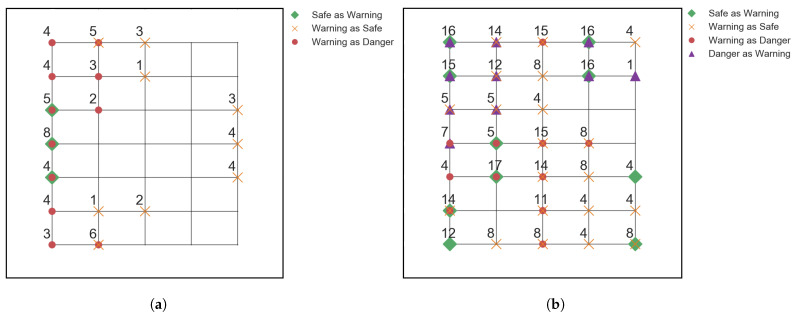
Locations of the misclassified impacts from the testing data for (**a**): 20% training data model, with 66/1008 misclassifications, and (**b**): 10% training data model, with 286/1134 misclassifications. The legends give information of the markers used, and the numbers next to the markers indicate the number of misclassifications on that location.

In order to assess the dependency of the Transformer to training data size, models were trained with different training data sizes, ranging from 10% to 80% of the overall available steel impactor data. For the tests, all the variables and parameters were kept the same, only the sizes of the training and testing data were changed accordingly. For each test, the F1 scores were calculated; the scores indicated that the predictive capabilities of the models start dropping fast with the decrease in training data samples. It was decided that the lower limit for accepting the model’s accuracy as exceptional be 0.99 F1 score. The 30% training data model was taken as the last instance of the Transformer achieving such high results, with 6 misclassifications out of 882 tests. To further examine the model’s accuracy below this 30% threshold and the misclassification variability in terms of location, the data from less accurate models, 20% and 10% training data, is taken, see Figure 6.

The grids are a representation of the grid for the impact locations in Figure 2. Each of the grid points corresponds to one of the impact locations L1–L35. The location data extracted from the less accurate models, along with the data from the best performing Transformer model, do not show a strong dependency on misclassification locations. The model obtains impacts wrong on a more random basis. This indicates that the Transformer model has not extracted features in a way that has developed a dependency on impact position. The model is therefore more robust, as the classification is uncorrelated with the position of the impact.

#### 4.1.2. CNN

The CNN is widely used for SHM and damage detection and classification. A range of papers exist that improve the CNN architecture and add to its performance. A 2-dimensional CNN model is build for this study with the intention of using its performance as a benchmark for the accuracy that a deep learning model can achieve with the available data.

**Figure 7:**
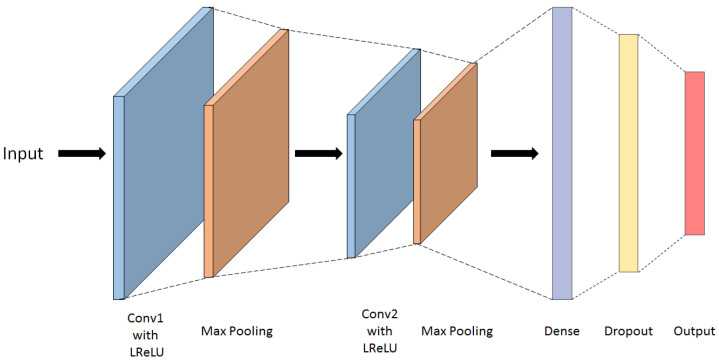
Architecture of the CNN framework used.

The data introduced to the network was kept the same as the one that was given to the Transformer model, namely the training data was taken to be 85% of the overall data, with the rest going to testing. The inputs were also kept consistent with the Transformer ones. Each test data was of the form 152 × 8 matrix for the Transformer, after the data transformation. For the CNN, the inputs were kept such, with the values being scaled between 0 and 1. The data points were scaled based on the maximum frequency amplitude value in the data set. This ensures that all inputs retain their shapes, relative one to another. This is crucial for the performance of the model, as it is able to learn not only on the shapes of the frequency spectra, but also on the relative energies each frequency bin carries. The CNN architecture that yielded the most accurate results for the steel data set is presented below in Figure 7. The number of kernels in the first and second Convolutional layers is 16 and 32, respectively, each with kernel size 3. Each of the two Convolutional layers is followed by a Max Pooling layer. A 32 neuron Dense layer follows, after which there is a dropout layer, of rate 0.1, to help with overfitting issues, and the output layer is again a Dense layer, with 3 neurons, as the number of classes is 3. All the activation functions used are Leaky ReLU, and the output normalization function is *Softmax*. Keeping consistency with the Transformer training, the trained CNN model was evaluated against the testing data (set aside from the steel impactor data), and the data from the silicone impactor tests. The F1 scores for the predicted data were 1 and 0.333 for steel and silicone, respectively, (see Appendix A, Figure A1).

**Figure 8:**
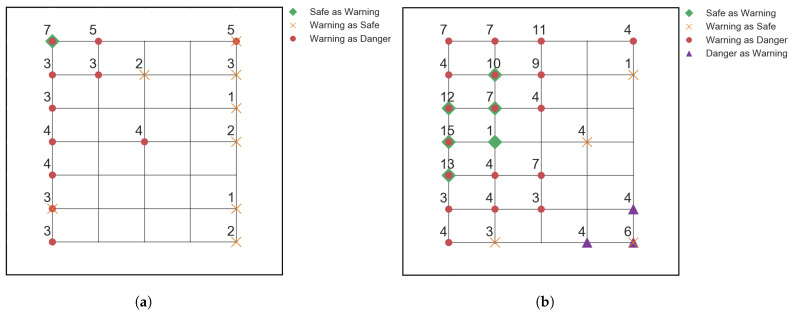
Locations of the misclassified impacts from the testing data for (**a**): 20% training data model, with 55/1008 misclassifications, and (**b**): 10% training data model, with 151/1134 misclassifications. The legends give information of the markers used, and the numbers next to the markers indicate the number of misclassifications on that location.

The network’s threshold of training data, before which it’s still very accurate, was investigated as well. The same CNN architecture was run with different training data sizes, ranging from 10% to 80% of the overall steel impactor data available, in steps of 10%. For consistency, all parameters, apart from the data splitting, were kept the same as the original test. The CNN was able to achieve perfect F1 scores with as little as 30%, 378 samples, of the available steel impactor data, while the performance decreased below this mark, the F1 scores were still high, around 0.8–0.95. A further investigation into what inputs the modes predicted wrong for the 20–10% interval was conducted, shown in Figure 8.

The small training set allows for the testing set to be large. Thus, the information extracted from the testing data, such as the misclassified positions, has higher accuracy. A slight dependence towards the model misclassifying impacts around the edges of the sensor grid can be observed on both tests. This can be explained as being due to the lack of data to create robust feature extraction. For impacts near the edges, the Lamb waves would be well detected by the nearest sensors, but would greatly disperse before reaching the furthest ones. With not enough data, the model misclassifies the dispersed signals for a higher energy impact. Most of the misclassified data is Safe as Warning and Warning as Danger. These errors are not as crucial, as further investigation would conclude that the damage is not as severe. The more dangerous misclassifications are Warning as Safe and Danger as Warning, as they downgrade the severity of the impact, which could lead to neglecting the problem with the structure.

### 4.2. Angled Impact Dependency Investigation

In this section, and the next, the scalability of the Transformer model is tested. It is now investigated whether the network can accurately predict inputs from angled impacts, if during training it had not seen any such examples. The CNN is also tested for comparison. The angled impact data was excluded from the steel data set and 85% was set for training. All other network parameters were kept consistent with the best Transformer and CNN models from the previous section, in order to achieve most accurate results. The models’ performance was tested against the angled data, which both networks had not seen during training. Neither model was able to predict the 45 degree impact data accurately. Both models labelled all tests as Safe, leading to an F1 score of 0.333. Neither model was able to go beyond the training data feature distribution presented to it. Both models require 45 degree impact signals to be present in the training data in order to accurately predict any new 45 degree impact signals.

### 4.3. Silicone Impactor Dependency Investigation

**Figure 9:**
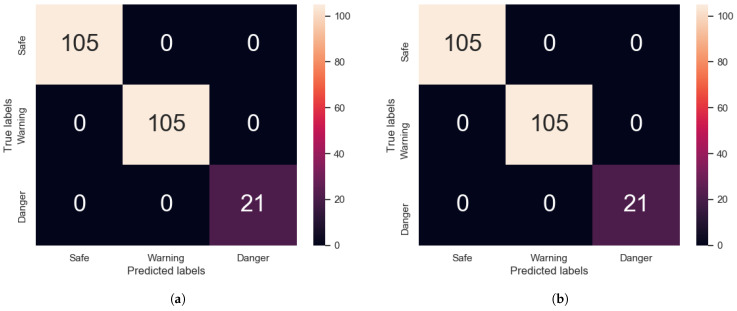
Confusion matrices for (**a**) Transformer and (**b**) CNN models, trained on steel and silicone impactor data together.

Both networks failed to accurately predict the silicone impactor data when they were trained only on steel data. It is now tested when the silicone data is introduced to the training data, whether the network will improve its accuracy on classifying silicone impacts. The silicone data was added to the overall steel data, with 85% of all the data going for training. The models were trained until convergence was reached. Their accuracy was tested against the testing data available, see Figure 9. In congruence with the previous tests, the training data was randomly selected from all the available data, but the ratios of samples of impact energy classes were kept the same as in the training set. The addition of the silicone data added two more test configurations, one labelled as Safe and one as Warning, making the overall number of unique test configurations present 11. The number of silicone impactor tests was 42, or 2/11 (18.18%) of all the testing data.

Both the Transformer and the CNN were able to accurately classify all the testing data. The Transformer was able to learn the new features from the silicone impactor data well enough to robustly classify all samples, once the silicone data was added to the training set.

## 5. Discussion and Comparison

This section compares the performances of the Transformer and CNN with the intention of evaluating the suitability of the Transformer as a valid way of detecting impact energies for composite panels. Summarizing comparison tables of the two models are presented in Appendix B.

### 5.1. Model Accuracy

The Transformer architecture performed with impeccable precision and achieved state-of-the-art performance when accuracy was measured, using the F1 score metric. The CNN, using the same amount of data, namely 85% for all steel data, was able to achieve 100% accuracy as well. The novel Transformer model can, with the highest degree of accuracy, determine whether an impact that has occurred during the life cycle of a composite part, is of little, medium or high severity. A system being able to assess this, instead of a human, cuts down maintenance time and costs drastically. It also allows for a more robust system to be in place, as a human operator is prone to errors.

The Transformer was able to retain its pristine accuracy up until the 30% training data mark for the steel impactor tests. Collecting impact data requires large amounts of effort, time and funds. The Transformer model allows for less data to be collected and introduced to the network, and still achieve 100% accuracy. This reduces the experimental work needed, saving time and money, without sacrificing accuracy. The CNN model too was able to retain its 100% accuracy of the testing data, even with very limited training data supplied. The lower limit for the model achieving pristine accuracy was, like for the Transformer, taken to be 30% of the original steel impactor data. Under this mark, the CNN achieved better results than the Transformer on both the 20% and 10% data, as can be seen from comparing Figure 6 and Figure 8. The better performance of the CNN can be attributed to the larger amount of work conducted for it. CNN architectures have been used extensively for more than a decade and have seen numerous improvements. Transformers were introduced only a few years before the writing of this paper. Little work has been conducted to make them more robust for the purposes of classification, yet the model’s performance compares to that of the CNN.

It is worth noting that both the Transformer and the CNN were able to achieve 100% accuracy on the Danger samples for the best performing models trained. The Danger samples are the most safety critical ones, as they are most likely to cause fibre fracture and delamination, or other sub-surface damage. This damage is much harder to detect and can cause catastrophic failure. Yet both networks were able to train, so as to pick out these signals. This shows the robustness of these models for real-world applications.

### 5.2. Scalability

The Transformer was not able to accurately classify data that sat outside its training data distribution. When the 45 degree steel impact data was excluded, the model was not able to properly classify it, yet when it was inside the training data, the model had no issues. The same was observed from the Transformer with the silicone data. The Transformer was able to merge the steel and silicone impactor signals under the same set of classes only once the silicone data was present in the training data. For both angled and silicone scalability tests, the model labelled all the *Safe* and *Warning* data as *Safe*.

The large differences between signals is thought to be the cause of the model’s inability to scale outside its training data range. In both scalability investigations, the Transformer’s training and testing sets differed in terms of input shapes. The 45 degree steel impact data signals induce Lamb waves with smaller amplitudes, compared to 90 degree steel impactor signals, due to the contact angle between the impactor and the plate. The silicone impactor signals differ even more greatly from the steel ones. Their amplitudes are much smaller and the signals are comprised mostly of low frequencies, compared to the more spiked behaviour of the steel impactor signals, as the softer silicone impactor material absorbs more of the impact energy, rather than it being transferred to the plate. Having learned the dependencies between features during training on impacts, which have produced larger Lamb waves, the Transformer naturally would label impacts, which have produced smaller Lamb waves, as less severe, mislabelling all as *Safe*. When given examples of these new data distributions during training, the Attention was able to adjust and properly classify them.

The CNN suffered from the same problems. It was only able to perfectly classify any data distribution, provided it had seen it before in its training data, as was demonstrated with the silicone and 45 degree angle data. In general, both the Transformer and the CNN were able to encompass new data into their feature extraction easily. Both models succeeded at generalizing and adjusting to new distributions, yet only when presented with examples during training.

### 5.3. Training Time

The training time for both network types was calculated for the most accurate models, the ones using 85% training data, with steel and silicone impactor data present during training. The most accurate models reached convergence in 150 and 110 epochs for the Transformer and CNN models, respectively, with the recorded times being Transformer: 54.4 s and CNN: 48.3 s. The training time for both models is very similar, with the CNN being faster solely due to needing less epochs until convergence. If epoch per time is taken, the Transformer is faster by close to 0.5 epochs/s, yet requires more epochs. A reason for this discrepancy can be attributed to the implementation of the network code. The CNN has guidelines on choosing and optimizing parameters. The Transformer model, still in its infancy for non-text applications, has not been developed as much. The Transformer’s marginally slower time of 54.4 s is a very fast time from a practical point of view, much lower than the time required for NDT. Thus, the Transformer is a time-effective method for impact classification.

### 5.4. Computational Demand

A network that is less demanding is industrially more appealing. In this category, the Transformer outperforms the CNN. To store the trained model weights, the Tensorflow Keras [43] save_weights method into an HDF5 file was used. The Transformer requires 445 KB of memory to store the model weights. In comparison, the CNN HDF5 file takes up 506 KB. This can easily be explained by looking at the number of trainable parameters for each of the two networks. The Transformer has 37,059 trainable parameters. The CNN has a total of 42,819 trainable parameters. The larger number of parameters results in more weights being updated, and later stored. For the same performance, the Transformer requires around 12% less storage than the CNN. For larger models, this storage space difference can become significant. The Transformer is more advantageous for aircraft applications, as storing it would require less space, compared to the popular CNN method.

The Transformer’s advantage manifests itself in two other areas. The memory usage of the Transformer during training is 1.2 GB. The CNN requires 1.9 GB. The Self-Attention method requires significantly less computational complexity than the Convolution method to reach the same performance. The CNN model would be more problematic to learn. On a powerful machine with parameters exceeding the aforementioned, a difference might be hard to spot. However, if a less capable machine is used, there will be a noticeable delay in the CNN training, while the computational demand during training is important for the process of tuning the model, once trained, the model’s practical usage is for performing predictions. For the memory usage during prediction, the Transformer uses 11.9 KB/prediction, compared to the CNN, with 15.3 KB/prediction. The notably lower memory usage allows for the trained Transformer model to be more versatile and to be ran in settings requiring lighter code.

## 6. Conclusions

A novel model for predicting impact energy for passive sensing in composite plates has been developed. The model, using a Transformer architecture, harnessing the Self-Attention method, was successfully developed and tested. A comparison with the well-known and widely used Convolutional Neural Network was conducted and insights about both networks’ performance were extracted. The main finding of the paper are listed below:

- Both the Transformer and CNN models are able to achieve 100% accuracy on impact energy classification, given steel impact signals.
- Both the Transformer and CNN were able to achieve highest accuracy with as little as 378 samples on steel data impactors. The further decrease in training samples deteriorated the Transformer’s performance on classifying Safe and Warning labels.
- The Transformer shows non-satisfactory up-scalability on new data sets. It is not able to accurately classify signals that are outside of the parameter distribution of its training set. The CNN equally struggles to predict data that lies outside its training samples.
- The Transformer is able to achieve pristine accuracy for any case when the training and testing data have the same distributions. The CNN equally achieves perfect prediction accuracy, when examples of the data it needs to predict have been present during its training. The two models are comparable on feature extraction and data generalization.
- The Transformer’s training time is approximately the same as that of the CNN, and it is much faster than the time required for NDT, making it a time-effective impact classification method.
- The Transformer, compared to CNN, requires much less computational power to train and run predictions. This makes it more flexible to be trained or executed on machines with less computational power, cutting costs from computational load.
- The Transformer requires 12% less memory space to be stored. This makes it more fit for aircraft applications where it would be implemented on-board, as aircraft free on-board memory is scarce. Even if not implemented on-board, the network saves memory space, cutting down costs.

The Transformer was able to achieve the highest accuracy, rivaling the generalization of features and accuracy of the established CNN model. It was marginally outperformed by the CNN in terms of speed of training. Considering the amount of research conducted for CNN and how novel Transformers are, the results show a great potential for the Transformer model. The Transformer proves to be a very promising network for the purposes of SHM, as it requires very little memory to be stored or ran. It is quick to converge, significantly cutting down maintenance time and costs. As a new network, it is expected that the following years will bring advancements in its performance, which will further make it appealing for aircraft impact classification and other engineering applications.

## Data Availability

Not applicable.

## Appendix A

### Appendix A.1. Transformer/CNN Validation Confusion Matrices

The confusion matrices presented below apply to both the Transformer and the CNN models, as they performed the same when trained on 85% of all steel impactor data, and then tested on the rest of the data (15% steel and 100% silicone impactor).

## Appendix B

### Appendix B.1. Model Comparison Table

**Table A1:** Summary comparison table between Transformer and CNN model performance for different training and testing conditions. The percentage of data used for training and testing the models is given in parentheses, where applicable. The F1 score metric is presented for the evaluation of the models’ performance for each test.

	Transformer	CNN
train: $90^{{}^{\circ}}$ steel + $45^{{}^{\circ}}$ steel (85%) test: $90^{{}^{\circ}}$ steel + $45^{{}^{\circ}}$ steel (15%)	1	1
train: $90^{{}^{\circ}}$ steel + $45^{{}^{\circ}}$ steel (85%) test: silicone	0.333	0.333
train: $90^{{}^{\circ}}$ steel (85%) test: $45^{{}^{\circ}}$ steel	0.333	0.333
train: $90^{{}^{\circ}}$ steel + $45^{{}^{\circ}}$ steel + silicone (85%) test: $90^{{}^{\circ}}$ steel + $45^{{}^{\circ}}$ steel + silicone (15%)	1	1

**Table A2:** Summary comparison table between Transformer and CNN model performance for training time and computational complexity.

	Transformer	CNN
Training Time [s]	54.4	48.3
Storage Memory [KB]	445	506
Training Memory Usage [GB]	1.2	1.9
Memory Usage per Prediction [KB]	11.9	15.3
